# A Possible Contraindication for Endoscopic Ultrasound-Guided Hepaticogastrostomy: A Giant Hiatal Hernia

**DOI:** 10.7759/cureus.66983

**Published:** 2024-08-16

**Authors:** Koichiro Mandai, Shiho Nakamura

**Affiliations:** 1 Department of Gastroenterology, Kyoto Second Red Cross Hospital, Kyoto, JPN

**Keywords:** hiatal hernia, endoscopic ultrasonography, drainage, contraindications, bile duct

## Abstract

We present the case of an 82-year-old female with obstructive jaundice secondary to a malignant distal biliary stricture. Endoscopic ultrasound-guided hepaticogastrostomy (EUS-HGS) was performed. The presence of a giant hiatal hernia induced dynamic liver movement during respiration, leading to unstable scope positioning. Despite the successful placement of a long, partially covered metal stent from the left intrahepatic bile duct to the intra-abdominal stomach, computed tomography performed three days later revealed free air and an increased distance between the liver and stomach. A subsequent endoscopy confirmed impending stent migration into the abdominal cavity, necessitating the insertion of an additional metal stent through the existing stent's mesh. The presence of a giant hiatal hernia may be considered a relative contraindication for EUS-HGS due to dynamic movements of the stomach and liver during respiration, which can cause stent migration, increased air leakage, and difficulty in establishing a stable fistula between these organs.

## Introduction

Contraindications for endoscopic ultrasound-guided hepaticogastrostomy (EUS-HGS) are similar to those for percutaneous transhepatic biliary drainage [1]. The presence of ascites can theoretically impair tract maturation [2], and most authorities suggest that a large amount of ascites is a contraindication for EUS-HGS because it can create a separation between the liver and stomach, preventing the formation of a mature fistula after the procedure. Additionally, there is a risk of peritonitis due to leakage of bile and intestinal contents [1,3]. Careful evaluation with cross-sectional imaging before EUS-HGS is crucial. Endoscopic drainage of the left intrahepatic bile duct in the presence of left lobe atrophy is not suitable and should be avoided [3]. Tumor infiltration of the gastric wall at the puncture site is also a contraindication due to the increased risk of bleeding [1,3]. However, data regarding other contraindications remains limited. Here, we describe a successful EUS-HGS procedure in a patient who subsequently developed postoperative adverse events due to a giant hiatal hernia.

## Case presentation

**Figure 1:**
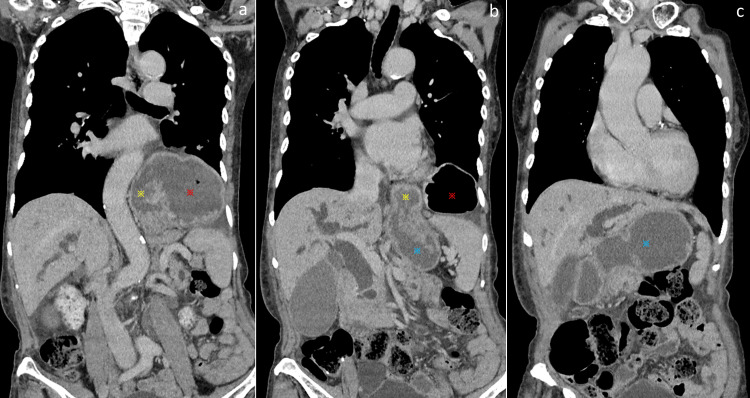
Computed tomography images a: the fundus (red asterisk) and proximal body of the stomach (yellow asterisk) are shown to have migrated into the thoracic cavity due to the giant hiatal hernia; b, c: the distal side of the gastric body (blue asterisk) is shown in the abdominal cavity.

An 82-year-old female presented to our institution with obstructive jaundice secondary to a malignant distal biliary stricture. Computed tomography revealed a giant hiatal hernia (Figure 1), making it difficult to insert a duodenoscope into the intra-abdominal portion.

**Figure 2:**
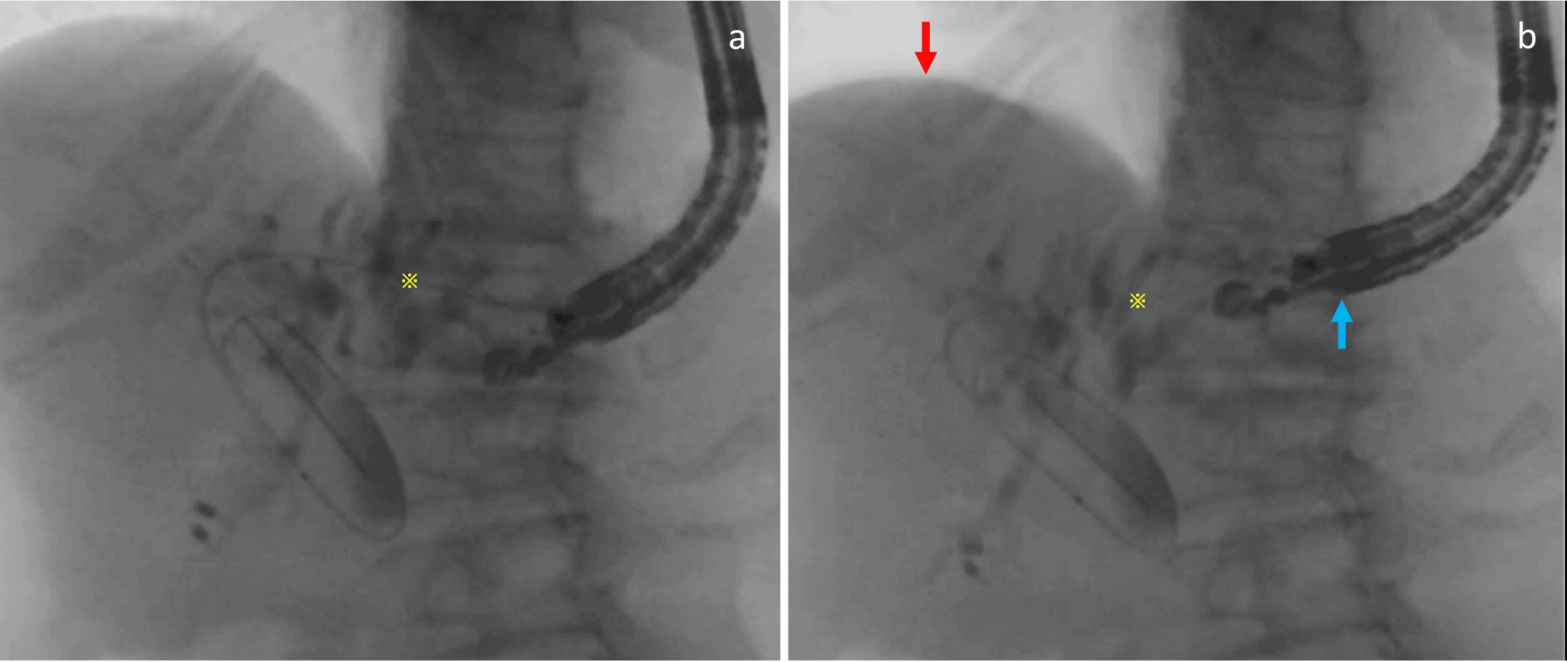
Fluoroscopic images Unlike expiration (a), the diaphragm pushes the liver caudally (red arrow) during inspiration (b), and the endoscope moves cranially (blue arrow) due to the giant hiatal hernia. The position relationship between the biliary puncture point (yellow asterisk) and the scope significantly differs during expiration and inspiration.

Therefore, single-balloon-assisted endoscopic retrograde cholangiopancreatography was attempted [4]. Although a duodenoscope was successfully inserted into the duodenum, biliary cannulation was impossible due to malignant duodenal stenosis around the papilla. Therefore, EUS-HGS was performed using the large balloon-assisted approach to facilitate endoscope navigation into the stomach [5]. The presence of a giant hiatal hernia caused dynamic movement of the liver during respiration (Figure 2), resulting in unstable scope positioning.

**Figure 3:**
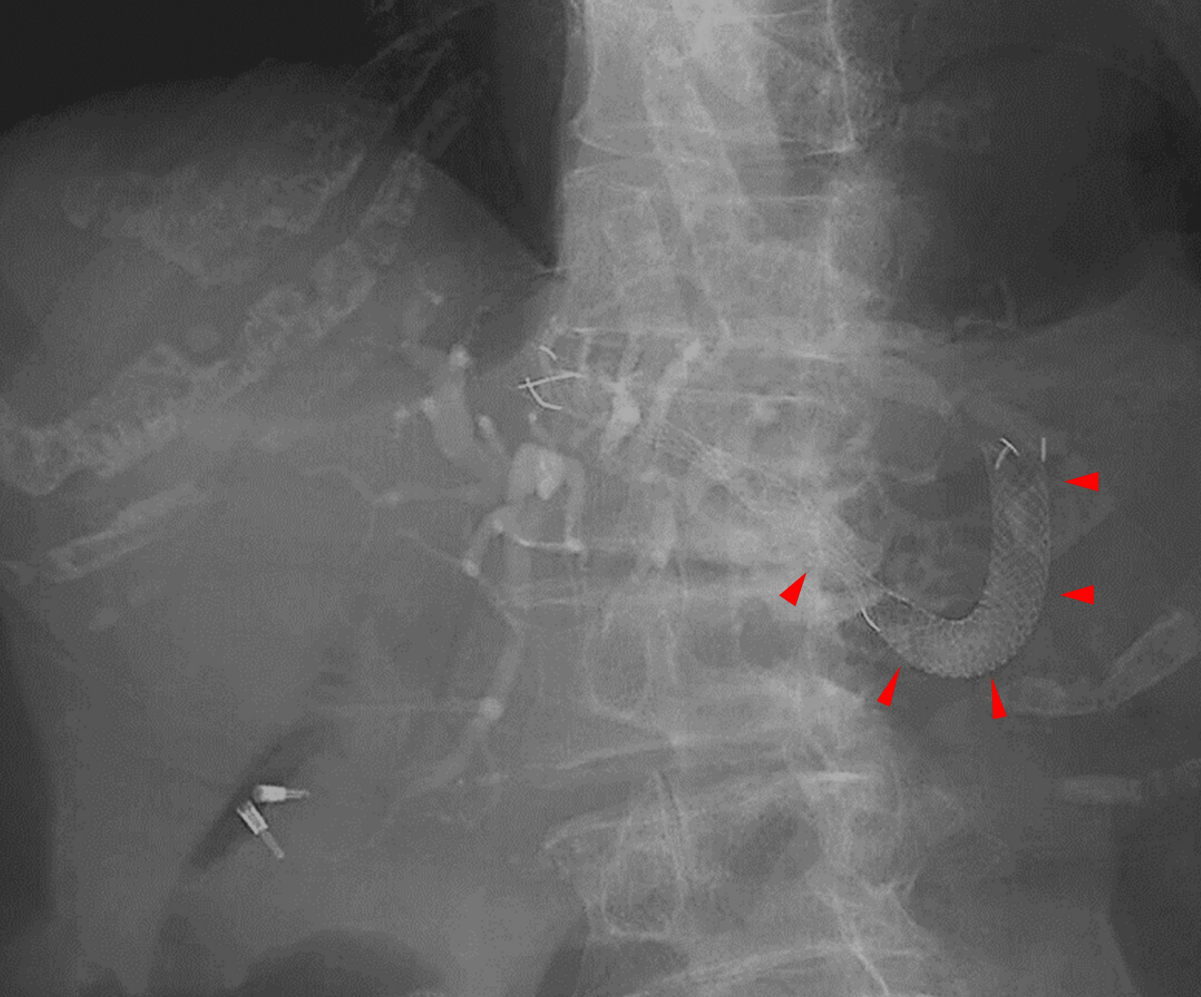
Fluoroscopic images At the time of stent placement, the intragastric stent length was sufficiently long (red arrowheads).

Consequently, we decided to complete the procedure as quickly as possible. As a result, we did not attempt to pass the guidewire through the distal biliary stricture, nor did we attempt antegrade stenting across the stricture. An 8 mm × 12 cm partially covered metal stent (Niti-S biliary S-type; TaeWoong Medical, Seoul, Korea) was successfully placed from the left intrahepatic bile duct to the intra-abdominal stomach. At the time of stent placement, the intragastric stent length was sufficiently long to prevent intraperitoneal migration (Figure 3).

**Figure 4:**
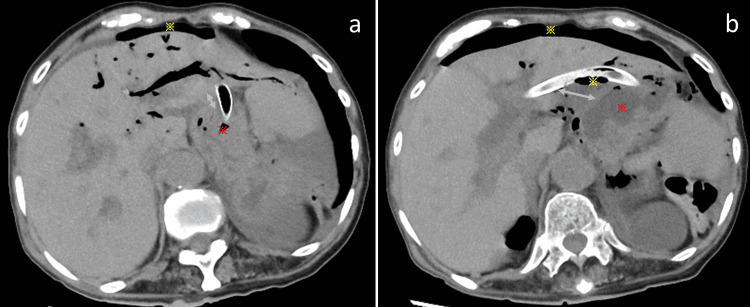
Postoperative images a: computed tomography on the following day reveals the presence of free air (yellow asterisk), although the liver and stomach (red asterisk) remain closely attached (double blue arrow); b: computed tomography performed three days later demonstrates an increase in free air (yellow asterisk) with a greater distance (double blue arrow) between the liver and stomach (red asterisk).

Although she was asymptomatic postoperatively, computed tomography (CT) on the following day revealed the presence of free air (Figure 4a). Subsequent CT performed three days later demonstrated an increase in free air, with a greater distance between the liver and stomach (Figure 4b). 

**Figure 5:**
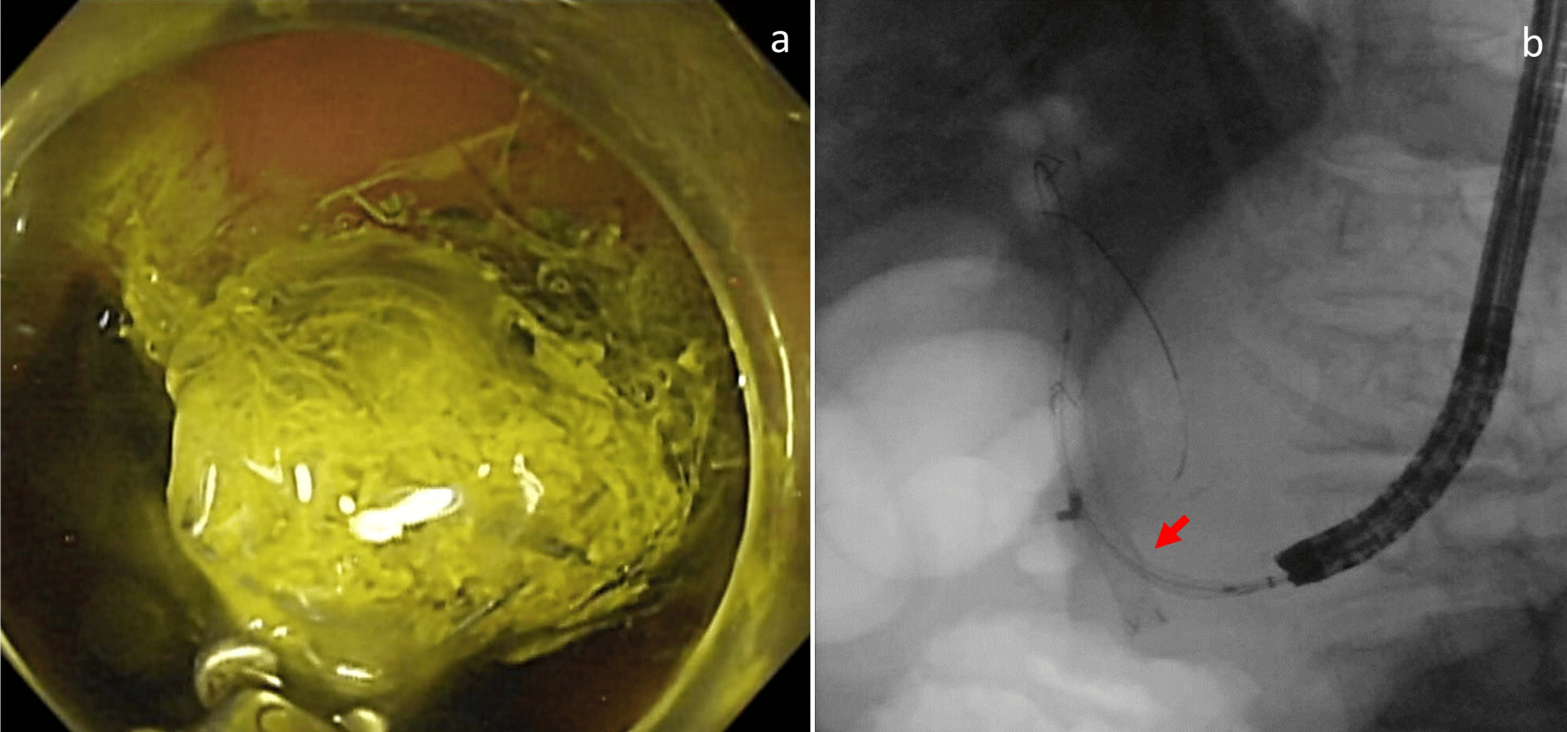
Images at the time of re-intervention a: endoscopy performed three days after endoscopic ultrasound-guided hepaticogastrostomy shows a short stent in the stomach that is on the verge of abdominal cavity migration; b: fluoroscopy shows the insertion of an additional metal stent through the mesh of the existing stent (red arrow).

Endoscopy confirmed impending stent migration into the abdominal cavity (Figure 5a), necessitating the insertion of an additional metal stent through the mesh of the existing stent (Figure 5b).

Although the patient's jaundice improved, she developed aspiration pneumonia and died seven days after EUS-HGS.

## Discussion

In cases of a giant hiatal hernia, such as an upside-down stomach, during inspiration, the liver moves caudally while the stomach ascends into the thoracic cavity through the enlarged esophageal hiatus (Figure 2). In the case we present, this dynamic movement caused impending stent migration into the abdominal cavity and increased air leakage from gaps between the stent and the gastric wall at the insertion site. This movement may also hinder fistula formation between the stomach and liver. Even if the liver and stomach are closely attached during the procedure, postoperatively, the stomach may gradually return to its original position, potentially shortening the length of the stent in the stomach and causing stent migration into the abdominal cavity [6]. Consequently, a giant hiatal hernia is considered a relative contraindication for EUS-HGS.

Alternative procedures

Percutaneous Transhepatic Biliary Drainage

This method carries no risk of stent migration into the abdominal cavity but has several disadvantages, including the risk of tube dislocation and potential patient discomfort leading to self-decannulation of the drainage tube.

EUS-Guided Transduodenal Biliary Drainage (EUS-Guided Choledochoduodenostomy)

The intra-abdominal distance of the stenting route is less likely to increase because the duodenum-common bile duct distance remains closer than the stomach-liver distance. However, inserting a convex scanning echoendoscope into the duodenum can be challenging in patients with a giant hiatal hernia [4,5]. Additionally, the presence of malignant duodenal stenosis near the papilla may complicate EUS-guided choledochoduodenostomy.

EUS-Guided Transgastric Antegrade Stenting Across the Stricture Without EUS-HGS

This approach eliminates the risk of stent migration into the abdominal cavity but poses risks of bile leakage through the puncture site and pancreatitis if the stent is placed across the papilla.

A recent study reported that the Spring Stopper, which has a spring-like anchoring function on the gastric side, is effective in preventing stent migration into the abdominal cavity [7] and may have been useful in this case as well. However, I believe that the dynamic movement of the liver and stomach during respiration would hinder fistula formation between these organs, even with the use of the Spring Stopper.

## Conclusions

The presence of a giant hiatal hernia may be considered a relative contraindication for EUS-HGS due to the dynamic movement of the liver and the stomach during respiration. These movements can lead to stent migration, increased air leakage, and difficulty in establishing a stable fistula between the stomach and the liver. The stomach may gradually return to its original position postoperatively, potentially causing stent migration into the abdominal cavity.

## References

[1] Isayama H, Nakai Y, Itoi T (2019). Clinical practice guidelines for safe performance of endoscopic ultrasound/ultrasonography-guided biliary drainage: 2018. J Hepatobiliary Pancreat Sci.

[2] Patel V, McLaughlin SW, Shlansky-Goldberg R (2019). Complication rates of percutaneous biliary drainage in the presence of ascites. Abdom Radiol (NY).

[3] Chantarojanasiri T, Ratanachu-Ek T, Pausawasdi N (2021). What you need to know before performing endoscopic ultrasound-guided hepaticogastrostomy. Clin Endosc.

[4] Sakai H, Iwai N, Okuda T, Sakagami J, Kagawa K (2021). Single-balloon-assisted ERCP in a patient with a giant hiatal hernia. Gastrointest Endosc.

[5] Itoi T, Watanabe H, Gotoda T, Tsuda H, Ootaka H (2015). Therapeutic endoscopic retrograde cholangiopancreatography using a large dilating balloon in a patient with upside-down stomach and bile duct stones (with video). J Hepatobiliary Pancreat Sci.

[6] Mandai K, Inoue T, Shinomiya R, Yoshimoto T, Ogawa T, Uno K, Yasuda K (2023). Safety of early oral intake after endoscopic ultrasound-guided hepaticoenterostomy. Surg Endosc.

[7] Ishii S, Isayama H, Sasahira N (2023). A pilot study of Spring Stopper Stents: novel partially covered self-expandable metallic stents with anti-migration properties for EUS-guided hepaticogastrostomy. Endosc Ultrasound.

